# Prevalence of pelvic organ prolapse in women, associated factors and impact on quality of life in rural Pakistan: population-based study

**DOI:** 10.1186/s12905-020-00934-6

**Published:** 2020-04-28

**Authors:** Abdul Hakeem Jokhio, Raheela Mohsin Rizvi, Christine MacArthur

**Affiliations:** 1 Department of Obstetrics & Gynaecology, Aga Khan University, Stadium Road, Karachi, Pakistan; 2 Institute of Applied Health Research, University of Birmingham, Birmingham, B15 2TT UK

**Keywords:** Community-based, Pelvic organ prolapse, Prevalence, Quality of life, Pakistan

## Abstract

**Background:**

Pelvic organ prolapse (POP) is a gynecological condition resulting from pelvic floor dysfunction in women. The objective of this study is to estimate “the prevalence of pelvic organ prolapse” associated factors, duration and impact on women’s quality of life in rural Pakistan.

**Methods:**

A cross-sectional study was conducted with a three stage random sampling strategy. Three health centers were selected and selected Lady Health Workers from each health center interviewed a random sample of women in their households. The interview used a structured questionnaire to collect symptom data. Female gynaecologists then conducted a clinical examination at the local health center on women who reported symptoms of prolapse to verify and grade pelvic organ prolapse using Baden-Walker classification system.

**Results:**

Among the 5064 women interviewed (95.8% response rate), 521 women had clinically confirmed POP, a prevalence of 10.3% (95% CI 9–11%). Among women with POP 37.8% had grade III or IV prolapse. Women with four or more children had the highest proportion of pelvic organ prolapse (75%) followed by women aged 36–40 years (25%).Among women with POP, 60.8% reported their quality of life as greatly or moderately affected; 44.3% had it for more than 5 years; and 78.7% never consulted a doctor.

**Conclusions:**

Pelvic organ prolapse is highly prevalent in rural Pakistan, impacts on women’s everyday lives and remains mainly untreated. Measures should be taken to provide health care services to reduce this burden of disease among women.

## Background

Pelvic Organ Prolapse (POP) is a common gynecological condition related to pelvic floor dysfunction in women [1]. It is the abnormal location of the pelvic organs, including the uterus, bladder, rectum or small intestine, into or outside the vagina [2]. It can result in surgery, which is one of the most common gynaecological surgical procedures performed with a lifetime risk of 11–19% in the general female population based on data from High Income Countries (HIC) [3, 4].

Much less is known about the prevalence and risk factors of POP from Low and Middle Income Countries (LMIC) [5]. A review of studies of pelvic floor disorders in LMICs published in 2011 found 13 studies with data on POP with prevalence estimates ranging from 3.4 to 56.4%, with a mean of 19.7%, however, most studies were small and not population based and had varying definitions and methods of ascertaining POP [6]. A population based study (*n* = 2070) not included in this review undertaken by UNFPA in Nepal found a prevalence of 10% based on asking the women if they had ‘something coming down in the vagina’ [7]. More recent studies from Ethiopia [8, 9] and Tanzania [10] showed prevalence ranging from 1% (based on symptoms included in general study of maternal health) up to 64.6% based on clinical examination.

Well established risk factors for POP, mainly based on data from HICs are older age [1], pregnancy and vaginal delivery, high parity and obesity [8], with others less consistently demonstrated including, early age at first delivery, forceps delivery, prolonged second stage of labour [10], carrying heavy objects or doing heavy work and high infant birthweight [10]. The distribution of these various risk factors differs in low and middle relative to high income countries, for example, high parity and younger age at first delivery being generally more common, whilst forceps delivery and caesarean section are less common. The rationale of our study was the lack of good data on POP and difficulties in assessing the burden of disease in women in LMIC’s including Pakistan. Since the rural areas are most neglected [10] hence studies from these areas warrants rigorous epidemiological investigation to assist in formulating strategies to provide appropriate services for treatment.

The objective of this study was to report the results of a large community population-based study, including gynecological examination, to investigate the prevalence of POP, risk factors and its impact on women’s quality of life, in rural Pakistan.

## Methods

The urinary and fecal incontinence and utero-vaginal prolapse (UFIUVP) study was a population based investigation of the prevalence of sub-types of pelvic floor disorders in women, their risk factors and its impact on women’s quality of life, to assess the burden of these diseases in a rural population in Pakistan. The aim was to assess the prevalence of the different disorders in a rigorous manner, including pelvic organ prolapse symptoms reported by women with signs confirmed by clinical examination.

The detailed study design, subjects, sample size, settings, data collection and other methods are described elsewhere [11, 12]. Using a simple systematic sampling scheme, at the first level half of the primary healthcare centers selected randomly, at the 2nd level 20% of lady health workers (LHWs) attached to these centers selected randomly, at third sampling level women aged 15 years and above and registered as households of each selected LHW were randomly selected.

A structured questionnaire (attached as an Additional file 1: appendix) was administered by trained Lady Health Workers (LHWs) through face to face home based interviews. Getting consent from the study participant was paramount and written informed consent was obtained from the subject, or from her parent or guardian of any participants under the age of 18 years. Demographic, socio-economic and the obstetric characteristic details were also recorded during the interviews. An appointment for a clinical examination was offered for symptomatic women.

The entry question to assess POP was; ‘do you experience a feeling of bulging or protrusion coming down from or in the vaginal area?’ (Yes /No). To enhance the understanding of the first question and likelihood of reporting, a second question was asked; ‘do you experience bulging or protrusion or something you can see in the vaginal area?’ (Yes /No). If the response to either question was positive, the woman was asked for further information to assess the stage of bulging or protrusion (comes and goes back at strain; partially out but need to push it up back into vagina; or completely hanging out from the vagina) and the duration and onset of symptoms. The women were asked “how much does bulging or protrusion bother you” to rate the impact of symptoms on their overall quality of life in general (not at all, slightly, moderately or greatly) (answer one only); and then they were asked “how much does bulging or protrusion interfere with your everyday life” to rate the impact on specific aspects of their everyday: hygiene, home life, work life and social life (not at all, slightly, moderately or greatly) (answer one only). Finally, they were asked if they had ever consulted a doctor because of their symptoms (Yes/No).

A urogynaecologist, expert in pelvic floor disorders and part of the study team, then reviewed all of the responses and categorized women as having POP (any of the symptoms of interest) or not. The women whose answers indicated the presence of POP symptoms were invited to attend the local health facilities to have a gynecological examination conducted by qualified local female gynecologists, experienced in clinical practice to verify and assign the diagnosis. These gynecologists received training to use the Baden-Walker classification system to asses POP because they found POP-Q staging system as cumbersome and complicated. The gynecologists took a standardized obstetric and gynecological history followed by clinical examination of the pelvic region and abdomen. A speculum and bimanual examination were performed with the patient in the left lateral position to assess the vaginal walls and cervix, asking the patient to perform maximum straining or coughing. The Baden-walker classification system was used to grade POP. It consists of four grades: grade 0 – no prolapse, grade 1 - halfway to Hymen, grade 2 – to hymen, grade 3 – halfway past Hymen, grade 4 –maximum descent. Women with POP were offered referral for clinical management.

Statistical analysis was conducted using SPSS software version 19.0 (IBM corporation Armonk, NY, USA). The prevalence estimates were calculated by considering those women in the numerator with POP verified by clinical examination as a proportion of all women interviewed.

Summary statistics were calculated using frequencies, percentages for categorical variables like grades of POP and means, medians, standard deviations and ranges for continuous variables such as age. The Chi-square test was used to evaluate relationship between the various categoricalvariables and factors associated with POP. Statistical significance was defined for *p*-values < 0.05.

The different factors independently associated with POP were analyzed using logistic regression after adjustment of confounding factors. Socio-demographic variables were then entered into logistic regression models. Odds ratios, *p* values and 95% confidence intervals were calculated for the relationships between the variables and the presence or absence of prolapse. No exposure was treated as main exposure for the POP (outcome) in this cross-sectional study, hence we did not have any confounder and as well as any plausible interaction for the study.

### Ethical approval

Ethical approval was granted by the Ethical Review Committee of Aga Khan University, Karachi, Pakistan, (vide no. 741-CHS/ERC-07, dated 27 June 2007).

## Results

**Fig. 1:**
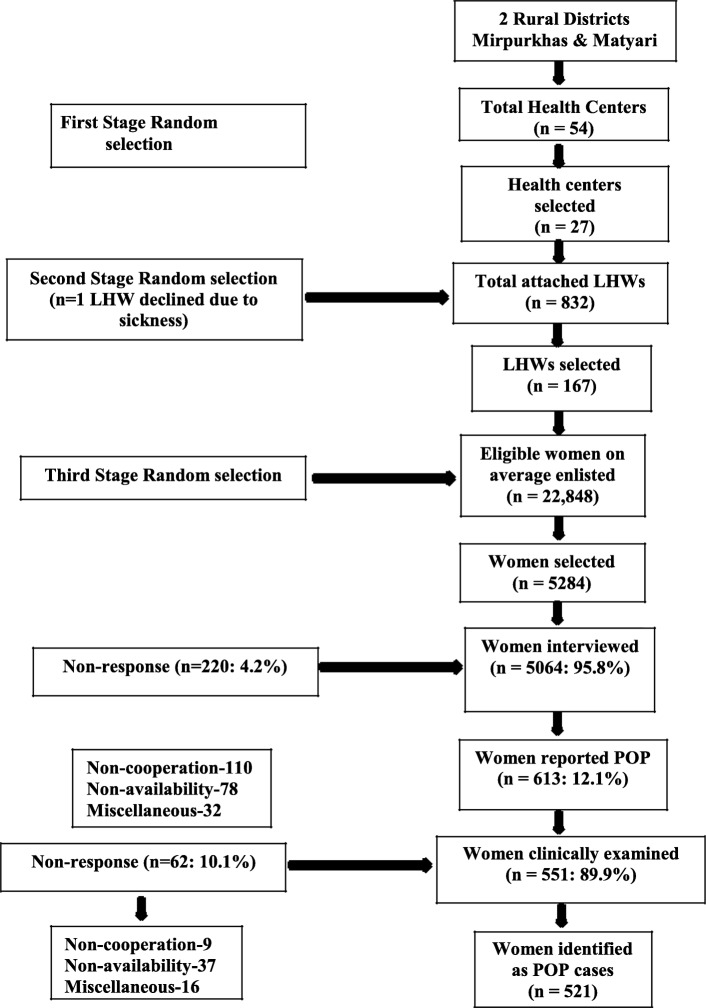
Diagram showing study flow

Of the 5284 women in the study areas approached to take part, 5064 were interviewed, a 95.8% response rate (Fig. 1). Of the 220 women not interviewed, 110 declined to take part and for 78 the LHW was unable to make an appointment after several attempts. There were 613 women who reported positively for POP in the questionnaire with different degrees of symptoms (see Table 1). Of these 613 women, 551 (89.9%) attended a clinical examination (Fig. 1). After clinical examination 30 (5.4%) women were not considered to have POP and 521 (94.5%) were verified as POP cases. These 521 were included as POP cases in the subsequent analysis. The prevalence of clinically verified POP in the sample was 10.3% (521/5064; 95% CI 9–11%).

**Table 1:** Symptoms of prolapse reported by women and grades of POP found on clinical examination.

Degree of prolapse/bulging		N 613*	%
1	Comes and goes back at the strain	261	42.6
2	Partially out but need to push it up back in vagina	188	30.7
3	Completely hanging out from vagina	164	26.8
**Women on which clinical examination conducted**			
**Baden-Walker system for evaluation of POP grade by clinical examination**		N 521**	**%**
1	Grade I (Descent halfway to hymen)	188	36.1
2	Grade II (Descent to the hymen)	136	26.1
3	Grade III (Descent halfway past the hymen)	89	17.1
4	Grade IV (Maximum possible descent for each site)	108	20.7

*All women who reported bulging/protrusion

**All women on which clinical examination conducted and found as POP cases

The grades of POP observed were: 188 (36.1%) grade-I; 136 (26.1%) grade II; 89 (17.1%) grade III; and 108 (20.7%) grade IV, (see Table 1). The type of POP within each grade is shown in Table 2, the most common being the cystocele grade I (*n* = 129, 24.8%). Anterior only genital prolapse occurred in 129 (24.8%) women; anterior and posterior in 131 (25.1%); posterior only in 48 (9.2%); and anterior, posterior and uterine in 213 (40.9%): there were no cases of anterior and uterine or posterior and uterine prolapse.

**Table 2:** Different types of pelvic organ prolapse according to grade of prolapse

Baden-Walker system for the evaluation of POP findings by clinical examination	N 521	%
**Grade-I of prolapse**		
Cystocele	129	24.8
Cystocele and Rectocele	59	11.3
Entrocele	0	
**Grade-II of prolapse**		
Entrocele and Rectocele	28	5.3
Rectocele and Cystocele	41	7.9
Cystocele, Rectocele and Uterine	67	12.9
**Grade-III of prolapse**		
Cystocele and Rectocele	31	6.0
Cystocele, Rectocele and Uterine	38	7.3
Rectocele and Entrocele	20	3.8
**Grade-IV of prolapse**		
Cystocele, Rectocele and Uterine	69	13.2
Cystocele, Rectocele, Entrocele and Uterine	39	7.5

Multivariable analysis showed that the only socio-demographic factors significantly associated with increased likelihood of POP were: increasing age (OR 1.05, CI 1.04–1.06) and higher parity (OR 1.18, 95% CI 1.15–1.21). Table 3 shows the individual age and parity groups, indicating a clear gradient for both, but no significant association between earlier age at marriage and POP. No significant association found with education, occupation, language spoken, religion and social class and POP.

**Table 3:** Odds ratios (95% Confidence Intervals) for selected socio-demographic associated factors with POP

Associated factor	Total women in group	Number with POP	Prevalence within group	Odds Ratio	(95% C.I)	P-value
**POP by age (in years)**						
15–20 yrs.	838	11	1.3	1.00	–	
21–25 yrs.	705	36	5.1	4.05	2.0, 8.0	< 0.001
26–30 yrs.	921	73	7.9	6.47	3.4, 12.3	< 0.001
31–35 yrs.	803	116	14.4	12.69	6.8,23.7	< 0.001
36–40 yrs.	814	130	16.0	14.29	7.7, 26.7	< 0.001
41–45 yrs.	414	56	13.5	11.76	6.1, 22.7	< 0.001
46–50 yrs.	286	48	16.8	15.16	7.7, 29.6	< 0.001
More than 50 yrs.	283	51	18.0	16.53	8.5, 32.2	< 0.001
**POP by parity**						
Para Nil	621	11	1.8	1.00	–	
Para 1–3	1861	108	5.8	3.42	1.8, 6.4	< 0.001
Para 4–6	1534	218	14.2	9.18	5.0, 16.9	< 0.001
Para 7 and more	1048	184	17.6	11.81	6.47, 21.9	< 0.001
**POP by age at the time of marriage (only married women included)**						
14 years or less.	337	43	12.8	1.00	–	
15–20 years.	3469	371	10.7	0.82	0.58, 1.15	0.24
21–25 years.	746	74	9.9	0.75	0.50, 1.12	0.16
26–30 years.	189	29	15.3	1.24	0.74, 2.06	0.41
31 years or more.	30	2	6.7	0.49	0.11, 2.12	0.34

**Results from binary logistic regression analysis.**

The duration of POP, its impact on the women’s overall quality of life, and consultation with a doctor about it are shown in Table 4. Among the women with POP, duration was long, with 44.3% reporting having had it for more than 5 years. In terms of their overall quality of life, 60.8% reported a moderate or great impact, while 47.8% reported that it moderately or greatly impacted their everyday life, including hygiene, home/work life and social life. Only 111 (21.3%) reported ever consulting any doctor about their POP. Among all women with POP, 91 (17.5%) also reported some form of urinary incontinence.

**Table 4:** Duration, impact on women’s overall quality of life and specific aspects of life of POP and consultation with a doctor

Variable	N	(%)
**Duration of POP: first began**		
3 to 6 months ago	28	5.4
7 months to 1 year ago	62	11.9
> 1 yr to 2 yrs. ago	68	13.1
> 2 yrs. to 5 yrs. ago	132	25.3
> 5 yrs. to10 yrs. ago	118	22.6
> 10 yrs. to 20 yrs. ago	80	15.4
> 20 yrs. ago	33	6.3
**Impact of POP on women’s overall quality of life**		
Not at all	80	15.4
Slightly	124	23.8
Moderately	149	28.6
Greatly	168	32.2
**Impact of pop on specific aspect of everyday life including hygiene, home life, work life and social life:**		
Not at all	74	14.2
Slightly	198	38.0
Moderately	130	25.0
Greatly	119	22.8
**Ever consulted any doctor because of POP**		
Yes	111	21.3
No	410	78.7

## Discussion

In this population-based study in rural Pakistan, the prevalence of POP based on clinical examination of symptomatic women was found to be 10.3% (95% CI 9–11%) among women aged 15 years or older. This prevalence is consistent with a population based study in Nepal [7] and a study in rural Ghana [13]. However, a previous study from Pakistan reported a prevalence of 19.1% [14], while another community based survey in rural Gambia reported a much higher rate of [10] 46%. Even much higher prevalence of 64.6% was reported from a recent rural Tanzania study.

Two other recent studies both from Ethiopia reported differing prevalence, one included 395 women who completed a questionnaire and 294 of these women had a clinical examination in the questionnaire symptomatic pelvic organ prolapse (do you have a feeling of bulging/pressure or something seems to be coming down through the vagina or do you have a visible mass protruding via the vagina) was 6.3% but when women were examined 55.1% had anatomical prolapse stage II-IV [8]. The other reported a much lesser symptomatic prevalence (experienced aprolapse uterus where you can feel part of the womb protruding outside of the vagina) of POP of 1% in a very large study of general maternal health experiences [9]. The variation in the estimation of prevalence of POP from 1 to 64.6% in different studies seen due to applying different definitions of POP diagnosis, applying different methods of POP classifications, inclusion of different age groups, and the studies are conducted in rural and urban areas as different cultures with different perceptions. For example, the study in Gambia, was based on interview and examination of women randomly selected from a rural community but criteria for the diagnosis of POP used was categories of cases such as mild-uterine prolapse into vagina; moderate-cervix visible at introitus and severe-uterine descent outside of introitus without using a validated classification system [15]. In the Tanzanian study [10] the median age of the women was 46 years which is higher than our study of 37.6 years [11]. In the smaller Ethiopian study the median age was 35 years, similar to our study age group but a different questionnaire was used on the basis of the questionnaire that was used in a HIC country USA [9]. Their clinical examination prevalence was much higher than ours at 55% though all women were offered clinical examination in this study compared with only symptomatic women in our study.

In our study, we used a clear definition of POP verified by physical examination using the “Baden-walker half way scoring system” and we found that 94.5% of women were verified as POP cases on gynaecological examination. There were a further 62 women among those who declined clinical examination and we don’t know what proportion of these would have had diagnosed POP. We found that 37.8% of women had POP of grade III or IV which requires surgical treatment [1, 6]and this is consistent with the population based study conducted in Nepal where 38% of women had grade three or four POP,managed by surgical intervention [7]. The mean age of the women with POP in this study was 37.6 years with a mean parity of 5.5 births. Increasing age and parity were identified as risk factors for POP in our study and this is consistent with other studies in LMICs [10, 13, 15–17] as well as in developed countries [1, 18, 19].

Almost half of the women had a duration of POP of more than 5 years and 6% of women had POP for more than 20 years. Overall more than 60% of women reported symptoms as greatly or moderately impacting their overall quality of life and almost half reported that their everyday life activities were also affected greatly or moderately. However, despite the long duration of POP and its effect on women’s lives, only 111 (21.3%) women had consulted a doctor about their condition. A study from rural Ghana showed that 35.3% of women with prolapse had sought treatment [13]. There are numerous possible reasons for this low rate of consultations in LMICs, including shyness [6], social stigma [13], lack of resources and high cost of care and accepting that prolapse is “normal” [15]. However, in Pakistan context women are unaware of medical facilities available for treatment, there is lack of resources and may be high cost of care in the country [20].

The strengths of this study are its sampling method, the very high response rate, and the robust method of data collection along with clinical verification and applying a well-defined Baden-Walker classification system. However, there are some limitations. We did not use a specific questionnaire to evaluate ‘impact on quality of life’; however, we developed our questionnaire based on different available and validated instruments (e.g., PFD120) and this was pretested in a pilot study [11]. POP associated factors were assessed only for demographic/socio-economic and the obstetric variables and we did not assess other risk factors such as women’s height, weight, body mass index or any heavy work or carrying heavy objects. The women who did not report symptoms were not invited for gynaecological examination and they may have had POP but not reported symptoms. There were also 62 women who had reported POP but did not attend a clinical examination due to their personal reasons and there is a possibility of missing out few number of POP cases.

## Conclusions

This large population-based study with high response rate and robust sampling and data collection methods has shown that POP was highly prevalent in a Pakistan rural population The data on effect on quality of life showed that this is a substantial problem for the women affected yet the majority of affected women did not seek medical care. Attention is required at the population level to improve the awareness and knowledge of the problem and efforts need to be made to minimize the social stigma by women reporting and provision of appropriate services and surgical management.

## Supplementary information

**Additional file 1: Appendix**. Study questionnaire

## Data Availability

The datasets used and/or analysed during the current study available from the corresponding author on reasonable request.’

## Abbreviations

POP: Pelvic Organ Prolapse
HIC: High Income Countries
LMIC: Low and Middle Income Countries
UFIUVP: Urinary and Fecal Incontinence and Utero-Vaginal Prolapse
UNFPA: United Nations Population Fund (United Nations Fund for Population Activities-An Organization)

## References

[1] Akeel NY, Gurland B, Hull T. Pelvic Floor Disorders Related to Urology and Gynecology. In Fundamentals of Anorectal Surgery 2019 (pp. 571–582). Springer, Cham.

[2] Haylen BT, Maher CF, Barber MD, Camargo S, Dandolu V, Digesu A, Goldman HB, Huser M, Milani AL, Moran PA, Schaer GN, Withagen MI (2016). An International Urogynecological Association (IUGA) / International Continence Society (ICS) joint report on the terminology for female pelvic organ prolapse (POP). Int Urogynecol J.

[3] Smith FJ, Holman CD, Moorin RE, Tsokos N (2010). Lifetime risk of undergoing surgery for pelvic organ prolapse. Obstet Gynecol.

[4] Løwenstein E, Bent O, Helga G (2015). Incidence and lifetime risk of pelvic organ prolapse surgery in Denmark from 1977 to 2009. Int Urogynecol J.

[5] Gunasekera P, Sazaki J, Walker G (2007). Pelvic organ prolapse: don’t forget developing countries. Lancet.

[6] Walker GJA, Gunasekera P (2011). Pelvic organ prolapse and incontinence in developing countries: review of prevalence and risk factors. Int Urogynecol J.

[7] UNFPA. Status of reproductive morbidities in Nepal, 2006 Kathmandu; UNFPA, (2006).

[8] Belayneh T, Gebeyehu A, Adefris M, Rortveit G, Awoke T. Pelvic organ prolapse in Northwest Ethiopia: a population-based study. Int Urogynecol J. 2019:1–9. 10.1007/s00192-019-04196-1 31853596

[9] Ballard K, Ayenachew F, Wright J, Atnafu H (2016). Prevalence of obstetric fistula and symptomatic pelvic organ prolapse in rural Ethiopia. Int Urogynecol J.

[10] Masenga GG, Shayo BC, Rasch V. Prevalence and risk factors for pelvic organ prolapse in Kilimanjaro, Tanzania: A population based study in Tanzanian rural community. PLoS One. 2018;25, 13(4). 10.1371/journal.pone.0195910 PMC5919002 29694427

[11] Jokhio A, Rizvi R, Rizvi J, MacArthur C (2013). Urinary Incontinence in Women in rural Pakistan, Prevalence, Severity, Associated factors and Impact on life. BJOG.

[12] Jokhio AH, Rizvi RM, Rizvi J, MacArthur C (2014). Prevalence of obstetric fistula: a population-based study in rural Pakistan. BJOG.

[13] Wusu-Ansah OK, Opare-Addo HS (2008). Pelvic organ prolapse in rural Ghana. Int J GynaecolObstet.

[14] Sajan F, Fikree FF. Does early age at marriage influence gynecological morbidities among Pakistani women? J BiosocSci. 2002; 34: 407–417. 10.1017/s0021932002004078 12117218

[15] Scherf C, Morison L, Fiander A, Ekpo G, Walraven G. Epidemiology of pelvic organ prolapse in rural Gambia. West Africa. BJOG 2002; 109: (4) 431–436. 10.1111/j.1471-0528.2002.01109.x 12013164

[16] Lien Y-S, Chen G-D, Ng S-C (2012). Prevalence of and risk factors for pelvic organ prolapse and lower urinary tract symptoms among women in rural Nepal. Int J Gynaecol Obstet.

[17] Fitchett JR, Bhatta S, Sherpa TY (2015). Non-surgical interventions for pelvic organ prolapse in rural Nepal: a prospective monitoring and evaluation study. JRSM Open.

[18] Gyhagen M, Bullarbo M, Nielsen TF, Milsom I. Prevalence and risk factors for pelvic organ prolapse 20 years after childbirth: a national cohort study in singleton primiparae after vaginal or caesarean delivery. BJOG. 2013 120(2):152–60). 10.1111/1471-0528.12020 23121158

[19] Glazener C, Elders A, MacArthur C, Lancashire RJ, Herbison P, Hagen S, Dean N, Bain C, Toozs-Hobson P, Richardson K, Mcdonald A, Mcpherson G, Wilson D (2012). Childbirth and prolapse: long-term associations with the symptoms and objective measurement of pelvic organ prolapse. BJOG.

[20] Hassan A, Mahmood K, Allah H. Healthcare System of Pakistan. IJARP. 2017 Oct;1: (4):170–173.

