# Supplementary material for: Prevalence of pelvic organ prolapse in women, associated factors and impact on quality of life in rural Pakistan: population-based study
Source: BMC Womens Health. 2020 Apr 28;20:82. doi: 10.1186/s12905-020-00934-6 (PMC7189438; doi:10.1186/s12905-020-00934-6)
Supplement: Supplementary file 1 — Additional file 1: Appendix. Study questionnaire [file 12905_2020_934_MOESM1_ESM.doc]

**Questionnaire**

**Part A (Socio-economic part)**

A1. Name of the woman_________________

A2. Husband’s name (if married) __________/Father’s name (If un-married) _________

A3. Caste ______________________________

A4. Age (in years) _________________

A5. Years of education______________________

A6. Occupation_________________

A7. Religion ________________________

A8. Language speak in home. ____________________

A9. Husband’s education (if married) _____/Father’s education (if un-married) _______

A10. Husband’s occupation (if married) ___/Father’s occupation (If un-married) _______

A11. Age (in years at the time of marriage _____ **(If un-married then go to Q no. ----)**

A12. Total Number of Children Born_____________

A18. Complete address: ____________________________________________________

________________________________________________________________________

**B-POP Questions**

| **Q. No** | | | **Questions** | **Code** | **Skip/Answer** |
| --- | --- | --- | --- | --- | --- |
| B1 | Do you experience a feeling of bulging or protrusion or coming down from or in the vaginal area? | | | 1. Yes  2. No |  |
| B2 | Do you experience bulging  or protrusion or something  you can see in the vaginal area? | | | 1. Yes  2. No |  |
| B3 | What is the degree of bulging or protrusion?  **(Check one answer.)** | | | 1. Comes and goes back at the strain  2. Partially out but you need to push it up back in vagina  3. Completely hanging out from vagina |  |
| B4 | When in your life did this bulging or protrusion FIRST begin as close as you can recall?  **(Check one answer.)** | | | 1. 3 to 6 months  2. 7 months to 1 year ago  3. More than 1yr to 2 yrs  4. More than 2yr to 5 yrs  5. More than 5yr to10yrs  6. More than10yr to 20yr  7. More than 20 yrs ago |  |
| B5 | How much does bulging or protrusion bother you?  **(Check one answer.)** | | | 1. Not at all  2. Slightly  3. Moderately  4. Greatly |  |
| B6 | How much does bulging or protrusion interfere with your everyday life?  **(Check one answer.)** | | | 1. Not at all  2. Slightly  3. Moderately  4. Greatly |  |
| B7 | | Have you ever consulted any doctor because of bulging or protrusion? | | 1. Yes  2. No |  |
| B8 | | If yes give details | | --------------------- |  |
